# Utilizing Nonpolar Organic Solvents for the Deposition of Metal-Halide Perovskite Films and the Realization of Organic Semiconductor/Perovskite Composite Photovoltaics

**DOI:** 10.1021/acsenergylett.2c00120

**Published:** 2022-03-03

**Authors:** Nakita K. Noel, Bernard Wenger, Severin N. Habisreutinger, Henry J. Snaith

**Affiliations:** † Clarendon Laboratory, Department of Physics, University of Oxford, Parks Road, Oxford OX1 3PU, U.K.; ‡ Princeton Institute for the Science and Technology of Materials, Princeton University, 70 Prospect Avenue, Princeton, New Jersey 08544, United States

## Abstract

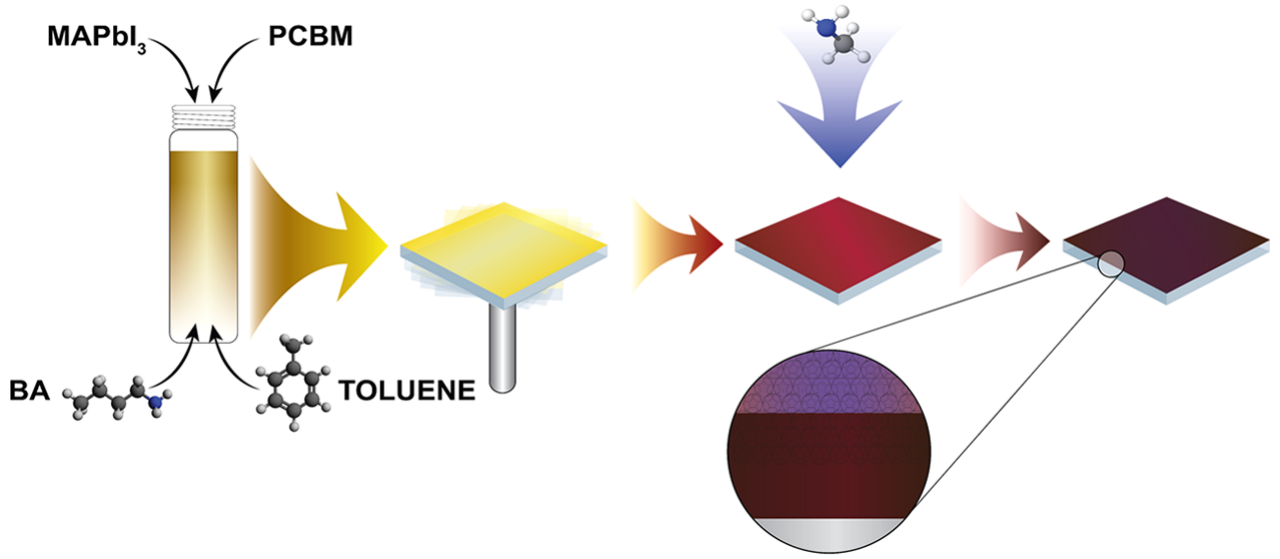

Having captivated the research community with simple fabrication processes and staggering device efficiencies, perovskite-based optoelectronics are already on the way to commercialization. However, one potential obstacle to this commercialization is the almost exclusive use of toxic, highly coordinating, high boiling point solvents to make perovskite precursor inks. Herein, we demonstrate that nonpolar organic solvents, such as toluene, can be combined with butylamine to form an effective solvent for alkylammonium-based perovskites. Beyond providing broader solvent choice, our finding opens the possibility of blending perovskite inks with a wide range of previously incompatible materials, such as organic molecules, polymers, nanocrystals, and structure-directing agents. As a demonstration, using this solvent, we blend the perovskite ink with 6,6-phenyl-C-61-butyric acid methyl ester and show improved perovskite crystallization and device efficiencies. This processing route may enable a myriad of new possibilities for tuning the active layers in efficient photovoltaics, light-emitting diodes, and other semiconductor devices.

Metal-halide perovskites are extremely promising photoactive materials, having been used to fabricate various optoelectronic devices such as solar cells, light-emitting diodes, and lasers.^1−5^ Photovoltaic devices based on perovskite materials are, to date, the most heavily researched of the halide perovskite-based applications, achieving certified power conversion efficiencies (PCEs) exceeding 25% on lab-scale single-junction cells, and just shy of 30% when combined with silicon in tandem cells.^6^ The ease with which these materials can be fabricated is one of their most attractive properties, with high-quality, crystalline layers being produced from simple solution-coating methods.^1,2,7−9^ One of the disadvantages of solution-processing these materials, however, and a substantial hurdle to overcome in the scale-up and commercialization of this technology, is the use of toxic, highly coordinating solvents with high boiling points, such as *N,N*-dimethylformamide (DMF). In fact, the use of DMF in the European Union will be further restricted in 2023, where for manufacturing or sale purposes the solvent cannot be used on its own and can only be used in mixtures where the overall concentration of DMF is <0.3%.^10^ This has significant implications for the feasibility of solution-processed perovskite optoelectronics and underscores the need for the perovskite research community to move away from and find alternatives to the often used 4:1 (v/v) DMF:dimethyl sulfoxide (DMSO) mixtures. Recent work by Vidal et al. has given a rigorous assessment of the impact of the most commonly used perovskite solvents on both human health and the environment.^11^ This study showed that DMSO had the lowest impact of all common perovskite solvents but, most importantly, provides a framework for assessing solvents based on the sustainability of their use, an important consideration as we move toward the commercialization of perovskite PV.

Further, we and others have previously discussed the chemical degradation pathways of DMF and have shown how this can affect the pH of the precursor solution. This then affects the eventual crystallization and chemical composition of the resultant perovskite films, adding further uncertainty in the parameters which control thin-film growth.^12−14^ Hence, in addition to the toxicity concerns associated with the use of DMF, the knowledge that the hydrolysis of this solvent directly affects the colloid distribution in perovskite precursor solutions, produces decomposition products which can be incorporated into the perovskite structure, and thus influences the optoelectronic properties of the films is an important consideration in the use of this solvent going forward.

Beyond considerations of industrially compatible solvents, an underexplored approach has been the ability to co-solubilize the perovskite precursors with a range of organic additives. If successful, this has the potential to enable much greater control over the crystallization process, allowing more uniform wet films to be achieved during solution coating. Furthermore, this would introduce new processing parameters which could be used to tune thin-film crystallization, including, but not limited to, the use of non-ionic surfactants or structure-directing agents. Being able to co-solubilize the perovskite salts with organic semiconductors could also enable a whole new range of hybrid organic–perovskite composites to be developed, including layered perovskite compounds with bulky organic semiconductor interlayers and organic–perovskite blended composite materials, akin to bulk heterojunctions. Attempts have been made to co-solubilize the perovskite precursors with polymers and small molecules, with varying degrees of success.^15,16^ The biggest hurdle to achieving this goal has, thus far, been the limited solubility of the organic material (organic small molecules or polymers) in the major perovskite solvent (DMF). The reduced solubility of organic molecules (e.g., phenyl-C61-butyric acid methyl ester, PCBM) and polymers (e.g., poly(methyl methacrylate), PMMA) in the perovskite solvents typically causes the organic species to precipitate out of the solution at relatively low concentrations.^15^ This, in turn, results in poor film formation during the crystallization of the perovskite thin film. In an attempt to circumvent this problem, the organic material has been added to the anti-solvent (either toluene or chlorobenzene) which is used to initiate the perovskite crystallization during the deposition process.^15−17^ Fortuitously, the anti-solvents most frequently used as “quenching” solvents during perovskite deposition are some of the most typically used solvents for organic small molecules and/or polymers. However, even in this case, there appears to be a threshold concentration beyond which the crystallization of the perovskite film is impeded. Additionally, this approach is unlikely to be scaled up into industrially relevant deposition procedures, which are unlikely to utilize the anti-solvent approach as is currently practiced in research laboratories. For blended perovskite–polymer/perovskite–small molecule films to be successfully deposited, it is imperative that both the perovskite precursors and the organic material have appreciable solubility in the parent solvent used in the ink formulation.

An obvious answer to this problem is to use a solvent in which both the perovskite precursor salts and the desired polymers/small molecules are soluble. However, this is not easily done, since the perovskite precursor materials are exclusively soluble in highly polar, aprotic solvents, whereas polymers/organic small molecules are typically soluble in nonpolar solvents. In previous work, we demonstrated that a compound solvent system can be used for perovskite thin-film deposition.^8^ Importantly, the host solvent is acetonitrile (ACN), a polar, aprotic solvent, which was combined with methylamine (MA) which acts as the primary solvent of the precursor materials. The addition of MA allows for the formation of a stable precursor ink, as MA gas is very soluble in ACN, to the point where enough of the amine is present in solution to keep the perovskite precursors dissolved. This is true for many polar, aprotic solvents such as acetone, butanone, and methyl ethyl ketone, for example.^8^ However, as the solubility of MA (a polar molecule) gas is much lower in nonpolar solvents, solvents such as toluene and chlorobenzene cannot be sufficiently saturated with MA gas to enable the dissolution of perovskite precursor materials at device-relevant concentrations. It follows, then, that one route to dissolving the perovskite precursors in nonpolar, organic solvents would be to utilize liquid amines which are miscible with the chosen host solvent.

In the present work, we show that, by using combinations of MA and butylamine (BA), we can both process the CH_3_NH_3_PbI_3_ perovskite material from nonpolar organic solvents and successfully deposit perovskite/PCBM blends. We also demonstrate a strong impact of PCBM concentration on the polycrystalline grain morphology and achieve improved photoluminescence (PL) lifetimes and photovoltaic (PV) performance, yielding steady-state PCEs exceeding 19% for both n-i-p and p-i-n device configurations.

First, we investigate whether the addition of longer chain alkylamines into the perovskite precursor solution has an impact upon the final perovskite formed. We use a standard 0.5 M solution of CH_3_NH_3_PbI_3_ in the ACN/MA compound solvent, as described in our previous work.^8^ Here, we use BA, as it is liquid at room temperature and is miscible with nonpolar solvents. By adding different volumes of BA to a full perovskite precursor solution, we find that we can tune the composition and structure of the perovskite from the 3D CH_3_NH_3_PbI_3_ to the layered (CH_3_(CH_2_)_3_NH_3_)_2_PbI_4_ material. Similar effects have been reported, where in lieu of a butylammonium iodide (BAI) treatment being used to form a 2D/3D interface with MA- or formamidinium (FA)-based 3D perovskites, BA is employed to obtain the same result.^18,19^ We show the X-ray diffraction (XRD) patterns alongside photographs of the films in Figure 1. From the XRD patterns, we observe that even with the addition of a very small amount of BA (10 μL/mL), a small peak appears at approximately 10°, which is indicative of the formation of an additional phase. At higher concentrations of BA (50 μL/mL) we see the appearance of another peak at approximately 27°. The emergence of these peaks is likely associated with the formation of mixed BA/MA Ruddlesden–Popper phases and can be matched very well to reflections from the *n* = 2 and *n* = 3 layered materials.^20^ Interestingly, when no MA is added to the dispersion and BA/ACN is used as the compound solvent, the XRD pattern of the resulting film matches exactly with that of the layered (*n* = 1) perovskite (CH_3_(CH_2_)_3_NH_3_)_2_PbI_4_.^20^ This suggests that when amines are used as solvents, there is an equilibrium between the solvent and the methylammonium cations, whereby (in this case) the BA solvent molecules can be protonated and thus incorporated into the crystal structure of the perovskite material.

**Figure 1:**
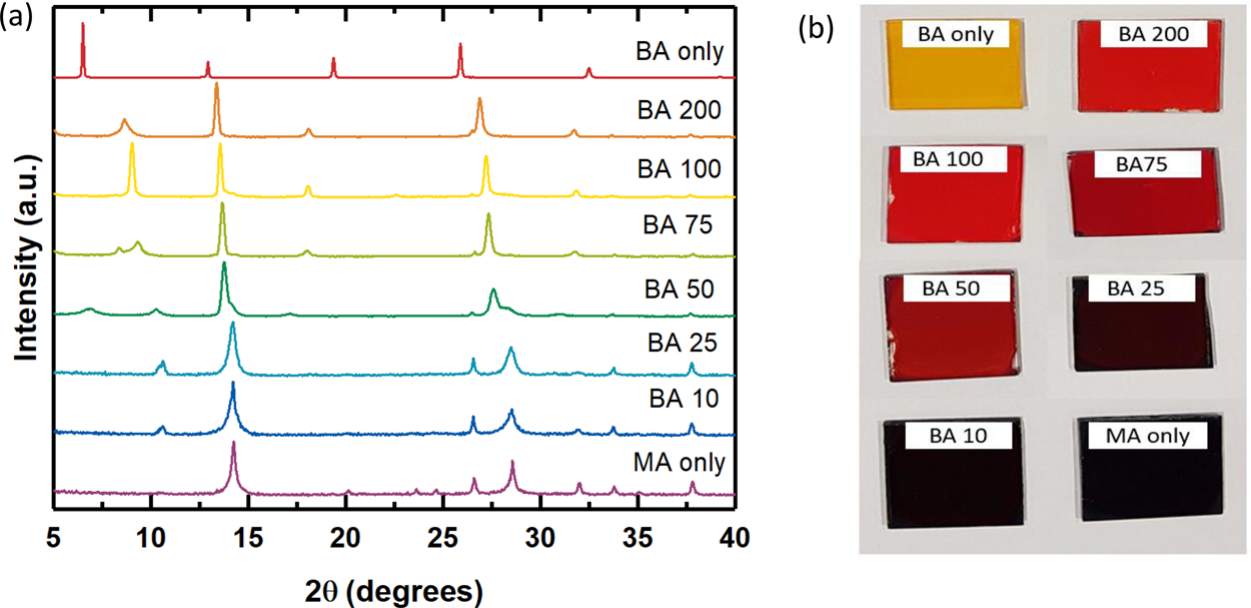
(a) X-ray diffractograms and (b) photographs of CH_3_NH_3_PbI_3_ films deposited from the ACN/MA compound solvent with various volumes of added BA. The legend and labeling denote the volume of BA in μL/mL of total solution volume. The overall molarity of all solutions was kept at 0.5 M. All films were annealed at 100 °C for 60 min.

We now assess whether BA can assist the dissolution of perovskite precursor salts in a non-solvent such as toluene. To minimize the amount of excess BA required in the perovskite solution, we first saturate the perovskite/toluene dispersion by bubbling MA gas into it for 10 min, after which we add BA to the dispersion until the perovskite is fully dissolved (full details are given in the Supporting Information). We then spin-coat the precursor ink onto the desired substrate and anneal for 60 min at 100 °C. In Figure 2a,b, we show the XRD pattern and UV–vis absorption spectra of such films. From the XRD traces in Figure 2a, we see that, in films processed directly from the MA:BA/toluene solvent mixture, as with the BA/ACN solvent mixture, the 2D BA_2_PbI_4_ perovskite material is formed. We confirm this by the absorption spectra shown in Figure 2b, showing the characteristic excitonic absorption displayed by this compound.^21^

**Figure 2:**
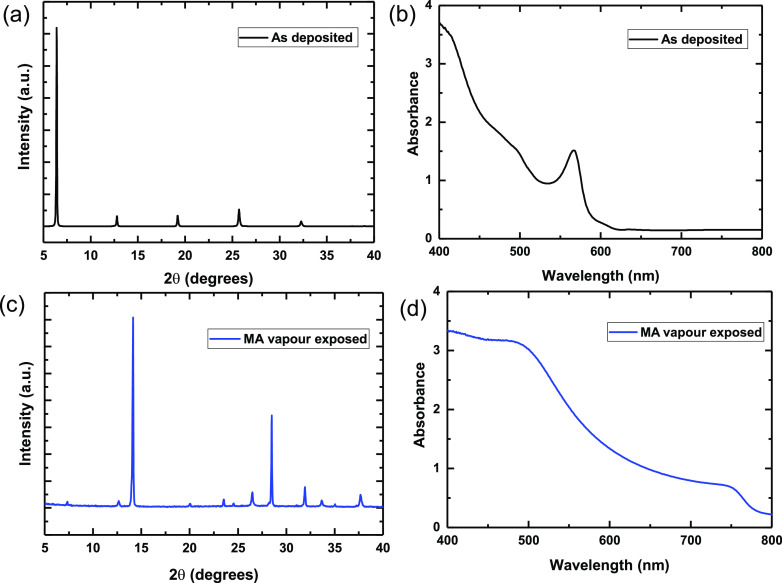
XRD patterns of perovskite films deposited from the MA:BA/toluene compound solvent before (a) and after (c) exposure to MA vapor. (b, d) Absorption spectra of perovskite films in (a) and (c), respectively.

This corroborates our initial hypothesis that long-chain alkylamines allow us to process halide perovskites out of nonpolar solvents. However, as in the case of using ACN as the polar host solvent, the solvating amines are incorporated into the perovskite crystal (as ammonium cations) and hence form a layered structure. Unfortunately, these layered structures have a comparatively wide bandgap, a non-negligible exciton binding energy, and limited charge transport, all of which make these layered materials less desirable for PV applications.^22^

It has previously been demonstrated that exposure of a perovskite film to MA vapor can not only lead to improved crystallinity and morphology but also cause changes in the composition of the material.^23,24^ For example, a film of dimethylammonium lead triiodide (at the time called HPbI_3_)^14^ which is exposed to MA vapor can be completely converted to CH_3_NH_3_PbI_3_.^25^ We therefore investigate whether this approach can also convert a butylammonium-based 2D perovskite film processed from the BA/toluene compound solvent into the 3D CH_3_NH_3_PbI_3_. After crystallization of the BA_2_PbI_4_ films, we expose them to MA vapor and subsequently anneal them for a further 15 min at 150 °C. In Figure 2c,d we show the XRD patterns and the absorption spectra of the films before and after MA exposure.

From the XRD pattern and corresponding absorption spectrum, we conclude that after we expose the film to MA vapor, the film appears to be almost completely transformed into the 3D CH_3_NH_3_PbI_3_, displaying its characteristic XRD peaks and absorption onset at 780 nm. We note the emergence of a small peak at approximately 7°, which we attribute to residual 2D perovskite remaining in the film. While we have used gas conversion to MAPbI_3_ here as an example for this proof-of-principle study, we note that the BA_2_PbI_4_ perovskite layers produced in this study can also be converted to FAPbI_3_ using our previously published protocols for converting MAPbI_3_ to FA-rich perovskites,^26^ combined with the higher annealing temperatures typical of FA-rich perovskites.^27−29^

Having shown that by using this sequential process we can successfully deposit CH_3_NH_3_PbI_3_ films from toluene, which is typically employed as an anti-solvent for the perovskite salts, we proceeded to investigate the utility of this solvent system for the co-deposition of the perovskite material and organic molecules; specifically investigating PCBM, a common component of perovskite solar cells. While PCBM is most frequently used as an electron extraction layer in perovskite-based solar cells, studies have also shown indications that it can lead to the apparent passivation of defect sites on the polycrystalline perovskite thin-film surface and at grain boundaries;^30,31^ however, the exact mechanism of this passivation remains unclear. Early works by Sargent and co-workers used small amounts of PCBM in the perovskite solution (resulting in PCBM being dispersed throughout the perovskite film) and showed increases in the PL lifetime of the films, while later studies obtained similar results through the inclusion of the molecule in the anti-solvent quenching step of film fabrication.^15,17^ Given that PCBM has an appreciably higher solubility in solvents such as toluene or chlorobenzene, we use the co-dissolution approach to investigate the impact of PCBM upon the optoelectronic properties and film formation. First, we investigate the influence of the addition of PCBM on the PL properties of the thin films. Figure 3a,b shows the steady-state and time-resolved PL of the films with increasing concentration of PCBM in the solution. We observe a monotonic increase in intensity of the PL, coupled with a slower decay rate, with increasing PCBM concentrations up to 10 mg/mL. As we increase the concentration of PCBM further, however, we see a monotonic decrease in the PL intensity and faster PL decays. By fitting the time-resolved PL decays to a stretched exponential function, we quantify the times taken for the PL to decay to 50% of the *t* = 0 intensity, which we term τ_50_. We obtain τ_50_ values of 291 ns for the control film where no PCBM is added to the precursor solution, which is comparable to the lifetime obtained for CH_3_NH_3_PbI_3-*x*_Cl_*x*_ films which have been processed from DMF.^32,33^ With the addition of low concentrations of PCBM to the precursor solution, we observe an increase in the PL lifetime, with a maximum of 1070 ns at 5 mg/mL of PCBM, after which it continually decreases to 44 ns at 20 mg/mL.

**Figure 3:**
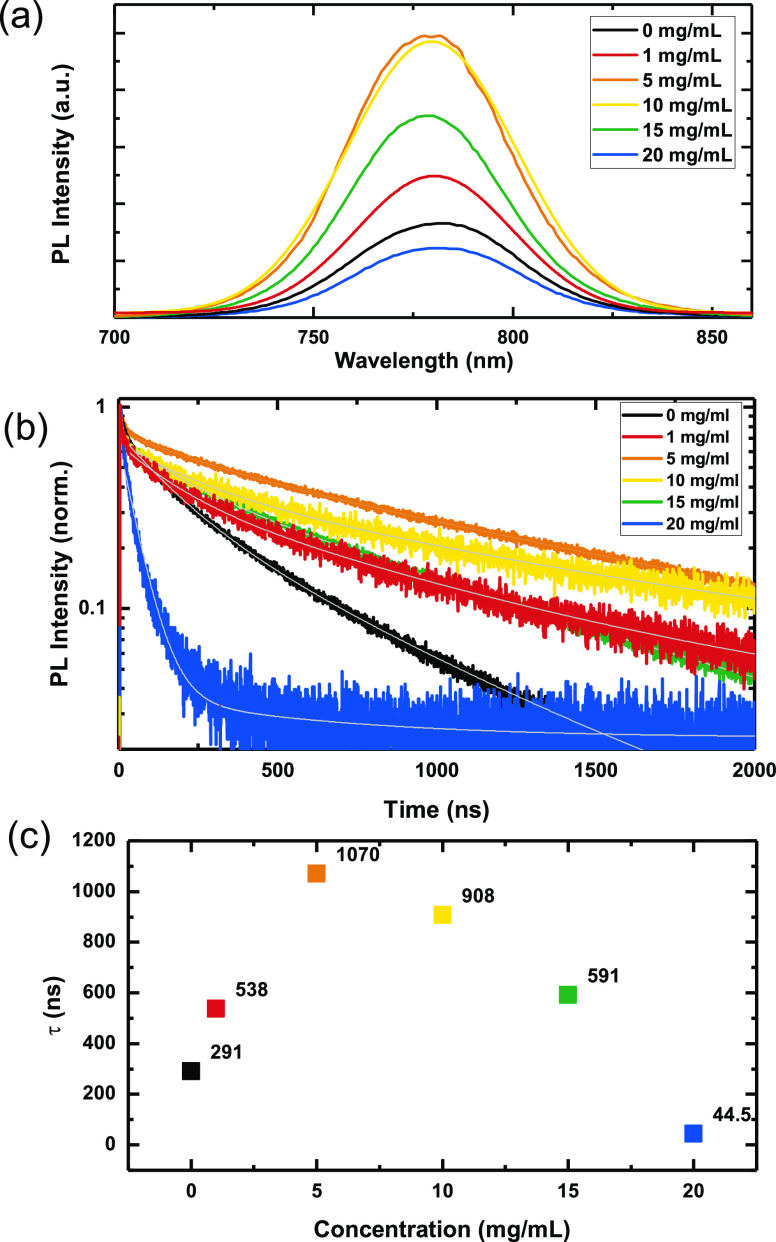
(a) Steady-state and (b) time-resolved photoluminescence of CH_3_NH_3_PbI_3_ films deposited from a BA/toluene compound solvent followed by MA vapor conversion, with specified amounts of PCBM added to the precursor solution. The solid gray traces are stretched exponential fits of the data. (c) Photoluminescence decay half-life times (τ_50_), as determined from the stretched exponential fits, as a function of PCBM concentration.

The initial increases in the PL lifetime and absolute PL are surprising, since PCBM is employed as an electron extraction layer in planar heterojunction cells and generally quenches the absolute PL and PL lifetime by a few orders of magnitude, either when processed on top of the perovskite or when the perovskite is processed on top of a layer of PCBM.^34^ However, our results here are consistent with literature reports where PCBM has been added at low concentrations into the DMF precursor solution^15,35^ or “infused” thermally down the grain boundaries from the top.^31^ These works suggest that PCBM may passivate defects at the surfaces and at the grain boundaries in perovskite films, resulting in the suppression of non-radiative recombination. Although theories have been espoused as to why this occurs,^15^ this apparent contradiction is unresolved, and there is no clear explanation as to why in some instances the presence of PCBM quenches the luminescence, and in other instances it enhances it. We now investigate the influence of PCBM addition on the film morphology and show scanning electron microscope (SEM) images of the resulting films in Figure 4.

**Figure 4:**
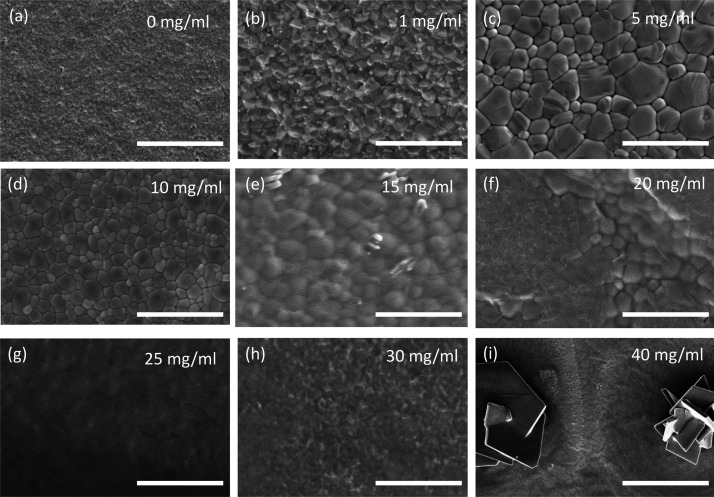
Top-view SEM images of CH_3_NH_3_PbI_3_ films deposited from an MA:BA/toluene compound solvent with subsequent MA vapor conversion, with increasing concentrations of PCBM dissolved in the precursor solution. All scale bars represent 5 μm.

From these SEM images, we see that the inclusion of PCBM into the precursor solution has a strong influence on the apparent polycrystalline grain size. At low concentrations, we observe a dramatic increase in the apparent grain size from sub-100 nm for the control films up to over 1 μm with approximately 5 mg/mL of PCBM added to the solution. At 20 mg/mL, we begin to see evidence of macroscopic phase separation of the perovskite and PCBM. In fact, there appear to be regions of crystalline perovskite materials surrounded or possibly covered with an apparent non-crystalline phase, conceivably PCBM. For PCBM concentrations of 25 and 30 mg/mL, the fine grain structure of the perovskite is no longer visible in the film, presumably due to it being covered by a continuous layer of organic material. However, at 40 mg/mL, we observe the appearance of significant clusters of very large crystallites and a very uneven distribution of material across the surface of the film. We note that at the highest concentration of 40 mg/mL, we are nearing the solubility limit for PCBM in toluene, and the precursor solution quickly becomes turbid when left to stand.

A change in the crystal quality of the perovskite films alone may be responsible for the increased PL lifetime, without having to invoke more complicated theories about interface passivation. However, we note the likelihood that at lower PCBM concentrations, the material is distributed throughout the film at grain boundaries, which may also lead to the “passivation” effect which we and others have observed.^15^ With increasing concentrations of PCBM, we see a decrease in the lifetime, with significant quenching occurring at 20 mg/mL. Looking at these results in the context of the changes in the apparent grain size and morphology of the films (as shown in the SEM images), we infer that at PCBM concentrations higher than 15 mg/mL, there is a substantial phase separation of the materials, with a PCBM layer forming on top of the perovskite layer as opposed to solely being distributed throughout the film. As PCBM and C_60_ have been shown to be extremely efficient extraction layers for perovskite solar cells,^36,37^ it is conceivable that at high concentrations, when a nearly complete layer of PCBM coats the perovskite, the PL lifetime is quenched as electrons are extracted from the perovskite and unfavorable surface recombination occurs.

Having assessed the optoelectronic quality of the films via PL measurements, we proceed to incorporate the films into solar cells. Here, we use the following device structure: FTO/SnO_2_/CH_3_NH_3_PbI_3_/spiro-OMeTAD/Ag. We show the performance statistics over four batches of devices in Figure 5 (steady-state efficiencies are given in Figure S1 in the Supporting Information). Most notably, we see an increase in the open-circuit voltage (*V*_OC_) of the devices where PCBM has been added to the precursor solution, with the maximum *V*_OC_ being achieved at 5 mg/mL of added PCBM. When the PCBM loading is increased beyond 10 mg/mL, we observe a sharp drop in all performance parameters. These results correlate well with the SEM images of equivalent perovskite films (Figure 4), where the film morphology deteriorates at higher PCBM loadings. From the SEM images, it appears that, at higher loadings (>15 mg/mL), more PCBM is present at the surface of the film. In the current n-i-p device architecture, this would result in direct contact with the spiro-OMeTAD layer, resulting in increased recombination at this interface and, hence, a drop in open-circuit voltage and an overall decrease in device performance, which is indeed what we observe in these devices. We show the current–voltage characteristics and steady-state power output traces of the control and best-performing devices in Figure 6.

**Figure 5:**
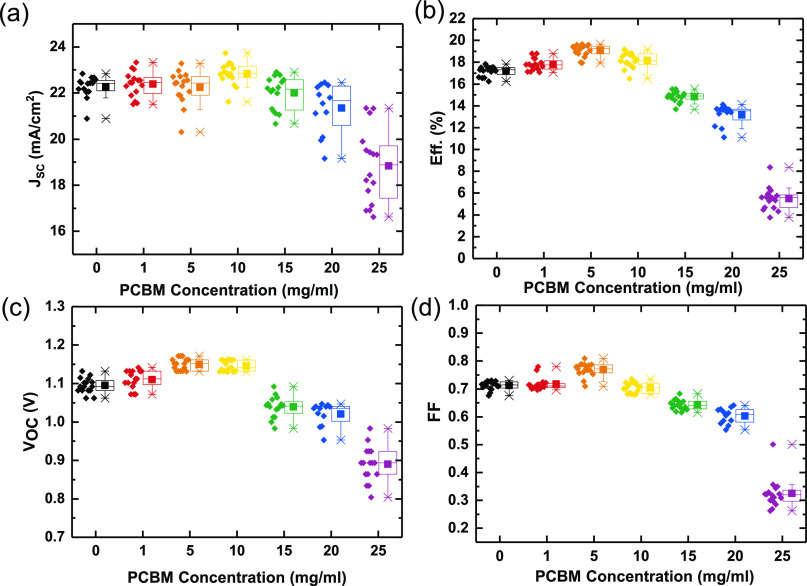
Performance parameters of CH_3_NH_3_PbI_3_ solar cells with specified concentrations of PCBM added to the precursor solutions. Each data point represents one device (total 16), made over four different device batches. Whiskers depict the 25th to 75th percentile, with points outside this range as outliers.

**Figure 6:**
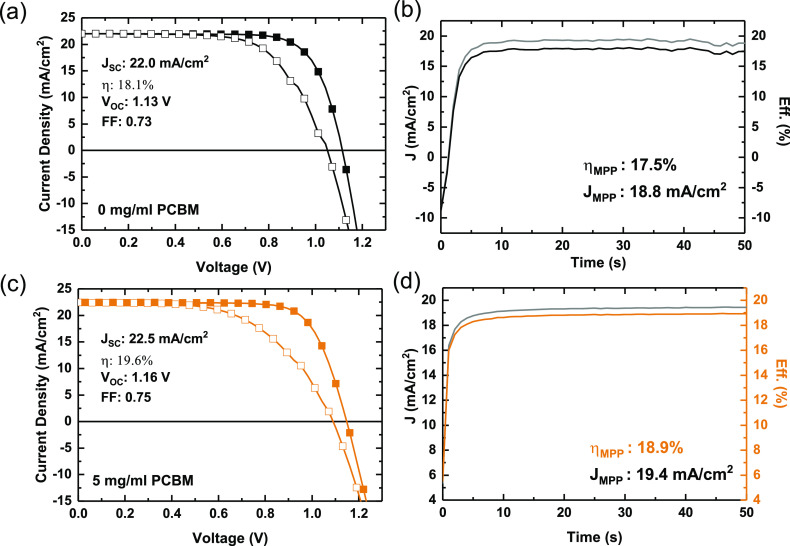
Current–voltage characteristics and steady-state efficiencies of champion CH_3_NH_3_PbI_3_ devices fabricated from a BA/toluene compound solvent, with and without PCBM additives.

We find that we achieve the best PCEs at PCBM loadings of 5 mg/mL, with the control and test devices yielding steady-state efficiencies of 17.5% and 18.9%, respectively. Notably, this is the PCBM loading at which we observe the largest apparent grain sizes and longest PL lifetime.

Having observed that, at higher concentrations, a phase-segregated layer of PCBM appears to form on top of the perovskite film, we consider the possibility that this spontaneous stratification may be advantageous in a p-i-n perovskite device architecture, where we intentionally require an n-type charge extraction layer on top of the perovskite film. To test this theory, we fabricate p-i-n-type solar cells with the architecture FTO/NiO/CH_3_NH_3_PbI_3_ + PCBM/C_60_/BCP/Ag, using a PCBM loading of 25 mg/mL in the precursor solution. At this PCBM concentration, the perovskite appears to be completely covered by PCBM, as we observe in Figure 3. To ensure complete coverage of the perovskite surface, we evaporate an additional 5 nm of C_60_ onto the perovskite:PCBM film, so as to plug any pinholes which may be present in the PCBM overlayer. A cross-sectional SEM of a full device is shown in Figure S2 in the Supporting Information. For our test devices, we achieve efficiencies exceeding 19%, which compares to less than 10% efficiency for the n-i-p devices fabricated with the 25 mg/mL PCBM loading (*J–V* curve of champion device shown in Figure S3). We show the current–voltage curves and steady-state efficiency of such a device in Figure 7.

**Figure 7:**
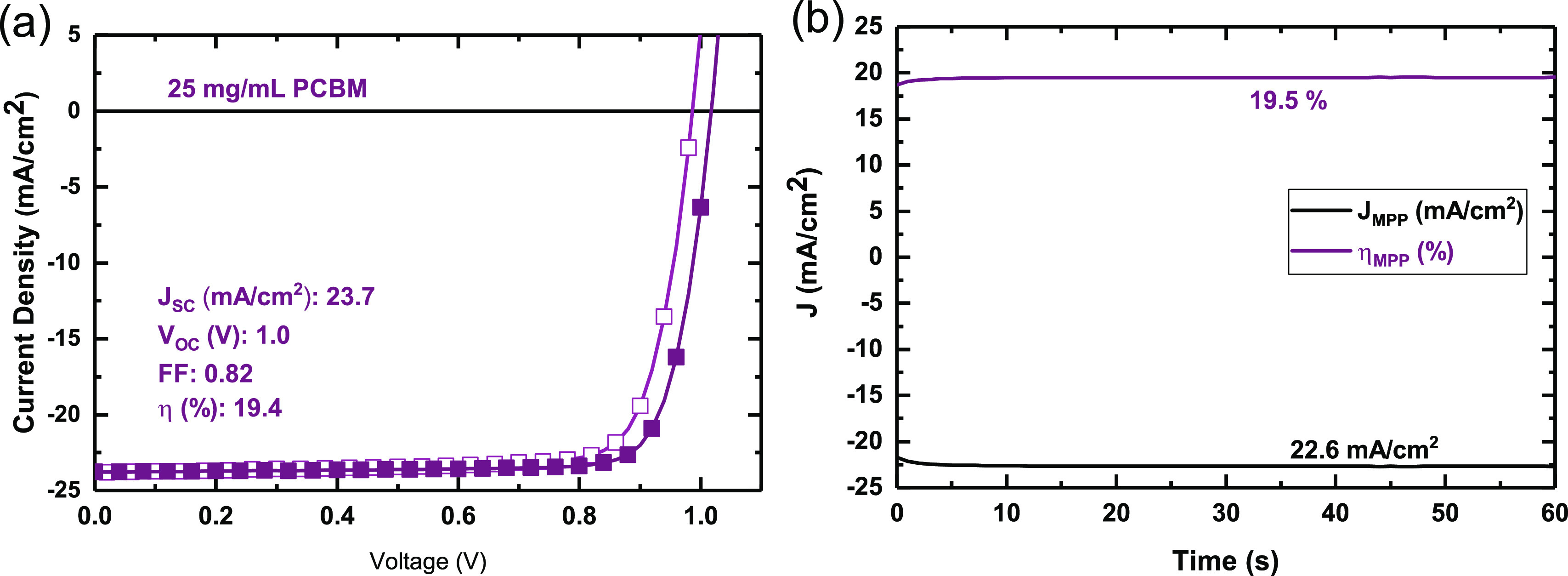
Current–voltage characteristics and steady-state efficiency of a prototypical p-i-n solar cell with 25 mg/mL of PCBM in the BA/toluene CH_3_NH_3_PbI_3_ precursor solution.

In summary, we have demonstrated a method by which the use of amine-based solvent systems can be extended to include nonpolar host solvents. This further relaxes the constraints on the possible solvents for the fabrication of perovskite solar cells, which could be an important development toward commercialization of this technology. Additionally, we show that using amines as a solvent for perovskite precursors is a simple method for the fabrication of the 2D-layered perovskite phases. By using a mixture of butylamine and methylamine in conjunction with the nonpolar organic solvent toluene, we are able to co-solubilize the perovskite precursors and the organic small molecule PCBM, allowing for co-deposition of the perovskite and organic charge transport materials. Incorporating these films into PV devices, we achieve maximum steady-state efficiencies of 17.5% for films deposited without PCBM and 18.9% for blended films. At higher PCBM loadings, we observe stratification of the films, where an overlayer of PCBM forms on top of the perovskite film. We take advantage of this property and construct p-i-n solar cells where an electron extraction layer is spontaneously formed on the perovskite surface and is conducive to efficient charge extraction. This yields solar cells with steady-state efficiencies of up to 19.5%. Here, we have demonstrated a unique strategy for solution-processing halide perovskite materials from a solvent blend of primary amines and organic nonpolar solvents. While the model system we have used for this demonstration is the perovskite solar cell, the ability to co-dissolve a range of small molecules and polymers opens a completely new space for the exploration of complex perovskite blend systems, conceivably with tunable optoelectronic properties and enhanced stability characteristics. This finding has the potential to benefit the entire field of perovskite-based optoelectronic devices, with possible applications beyond just photovoltaics.

## Supporting Information Available

Full experimental methods, device statistics, cross-sectional SEM, and current–voltage curves (PDF)
