# Supplementary material for: Utilizing Nonpolar Organic Solvents for the Deposition of Metal-Halide Perovskite Films and the Realization of Organic Semiconductor/Perovskite Composite Photovoltaics
Source: ACS Energy Lett. 2022 Mar 3;7(4):1246–54. doi: 10.1021/acsenergylett.2c00120 (PMC9084604; doi:10.1021/acsenergylett.2c00120)
Supplement: Supplementary file 1 — nz2c00120_si_001.pdf [file nz2c00120_si_001.pdf]

Utilising Non-Polar Organic Solvents for the Deposition of Metal-Halide Perovskite films and the Realisation of Organic Semiconductor/Perovskite Composite Photovoltaics

Nakita K. Noel,[1,2] Bernard Wenger,[1] Severin N. Habisreutinger,[1] Henry J. Snaith[1]*

[1] Clarendon Laboratory, Department of Physics, University of Oxford, Parks Road, Oxford, OX1 3PU, UK

[2] Princeton Institute for the Science and Technology of Materials, 70 Prospect Ave., Princeton, NJ, USA, 08544

Corresponding Authors: henry.snaith@physics.ox.ac.uk; nakita.noel@physics.ox.ac.uk

Supplementary Information

Preparation of Precursor Solution:

Methylammonium iodide (Dyesol) and PbI_2 (TCI Chemicals) were added to 3 ml of toluene in a 1:1 molar ratio, such that a 1M solution was obtained. The vial was sonicated until a dark yellow-grey suspension was obtained. The dissolution of the precursors was carried out in a modified version of a previously published methodology.[1] Briefly, a solution of methylamine (MA) in ethanol (Sigma Aldrich, 33 wt%)was placed into an aerator which was kept in an ice bath. A carrier gas, N_2, was then bubbled into the solution, thus degassing the solution of MA. The MA gas which was produced was then passed through a drying tube filled with a desiccant (Drierite and CaO), before it was bubbled directly into the toluene (Sigma Aldrich) which contained the perovskite precursors. The gas was bubbled into the dark grey dispersion for 5 minutes after which 500 µl of butylamine (Sigma Aldrich) was added to the dispersion. Upon addition of the butylamine, a clear, yellow solution was obtained, after which toluene was added to the solution such that the final molarity of the perovskite solution was 0.5M. PCBM was then added to the precursor solution in the desired quantity, and the solution was stirred at room temperature until the PCBM was completely dissolved.

Deposition of Perovskite Films:

The perovskite films were deposited onto the desired substrate by spin coating at 2000 rpm for 45 seconds in dry air, resulting in the crystallisation of a yellow film during spin coating. The films were then annealed at 100 °C for 60 min, after which it was allowed to naturally cool down to room temperature. When the substrate was completely cool, the films were held in methylamine vapour for 10 seconds, causing a bleaching of the perovskite films.[2] The films were then removed from the vapour and immediately placed on a hotplate after which they were annealed for a further 15 minutes at 150 °C, resulting in the formation of a $CH_3NH_3PbI_3$ perovskite film.

Optical Characterisation:

Absorption spectra were recorded on a Cary 3000 Uv-Vis spectrophotometer. Time-resolved PL measurements were acquired using a time-correlated single photon counting (TCSPC) setup (FluoTime 300 PicoQuant GmbH). Film samples were photoexcited using a 505 nm laser head (LDH-P-C-510, PicoQuant GmbH) pulsed at a frequency of 500 kHz, and fluence of 0.187 nJ/cm^2. The PL was collected using a high-resolution monochromator and hybrid photomultiplier detector assembly (PMA Hybrid 40, PicoQuant GmbH).

Device Fabrication:

Planar heterojunction solar cells were fabricated utilising previously published methods.[1,3]

n-i-p devices: Concisely, FTO-coated glass sheets (7 Ωcm^{-1} Hartford Glass) were etched with zinc powder and HCl (3M) to obtain the required electrode pattern. The sheets were then washed with soap (2% Hellmanex in water), deionised water, acetone, ethanol and isopropanol, and finally treated under oxygen plasma for 10 min to remove the last traces of organic residues. A compact layer of SnO_2 was then deposited on the glass using a modified version of the methodology presented by Anaraki et al.[4,5] Briefly, $SnCl_4.5H_2O$ was dissolved in isopropanol at a concentration of 0.05 M and stirred at room temperature for 30 min. The solution was then spin coated onto the substrates at 3000 rpm, after which the substrates were annealed at 180˚C for 1 hour before being left to naturally cool down to room temperature. The substrates were then immersed into a chemical bath, which consisted of $SnCl_2{\cdot}2H_2O$ (Sigma-Aldrich) in deionised water (0.012 M),

20.7 mM urea (Sigma-Aldrich), 0.15 M HCl (Fisher Scientific) and 2.87 μM 3-mercaptopropionic acid (Sigma-Aldrich). The substrates were kept in an oven at 70 °C for 180 min, after which they were sonicated in deionised water for 2 minutes. They were then washed with ethanol and annealed at 180 °C for 60 min. The perovskite films were then deposited as mentioned above. A spiro-OMeTAD layer was subsequently deposited using 85 mg/mL of 2,20,7,70-tetrakis(N,N-di-pmethoxyphenyl-amine)9,9-spirobifluorene (Lumtec) was dissolved in chlorobenzene (Sigma-Aldrich), with 80 mM Li-TFSI and 33 μL/ml of 4-tert-butylpyridine (Sigma-Aldrich) added. The solution was deposited onto the desired substrate spin coating at 2000 rpm for 45 s. The devices were then left in a desiccator in the dark for 12-16 hrs prior to electrode deposition. Finally, 120 nm thick silver electrodes were deposited under high vacuum (10^{-6}) in a thermal evaporator using a shadow mask.

p-i-n devices: 200 mg of 200 mg nickel acetate trihydrate (Sigma Aldrich) were dissolved in 4 mL anhydrous ethanol with 49 μL of ethanolamine. The solution was stirred overnight at 50 °C before being spin-coated onto cleaned (as above) FTO substrates at 3000 rpm for 45 s. The substrates were then transferred to a hotplate and annealed at 400 °C for 45 min before being allowed to cool down to room temperature naturally. When the substrate was cool the perovskite film was deposited and annealed as described above. 5 nm of C60 were then thermally evaporated under high vacuum followed by 1.2 nm of bathocuproine. Finally, 120 nm thick silver electrodes were deposited under high vacuum (10^{-6}) in a thermal evaporator using a shadow mask.

Current-Voltage Characterisation:

Solar cell performance was measured using a class AAB ABET sun 2000 solar simulator that was calibrated to give simulated AM 1.5 sunlight at an irradiance of 100 mW/cm^2. The irradiance was calibrated using an NREL-calibrated KG5-filtered silicon reference cell. Current-voltage curves were recorded using a sourcemeter (Keithley 2400, USA). All solar cells were masked with a metal aperture that was used to define the active area of the devices, which was 0.0925 cm^2 for n-i-p devices and 0.1 cm^2 and for p-i-n devices.

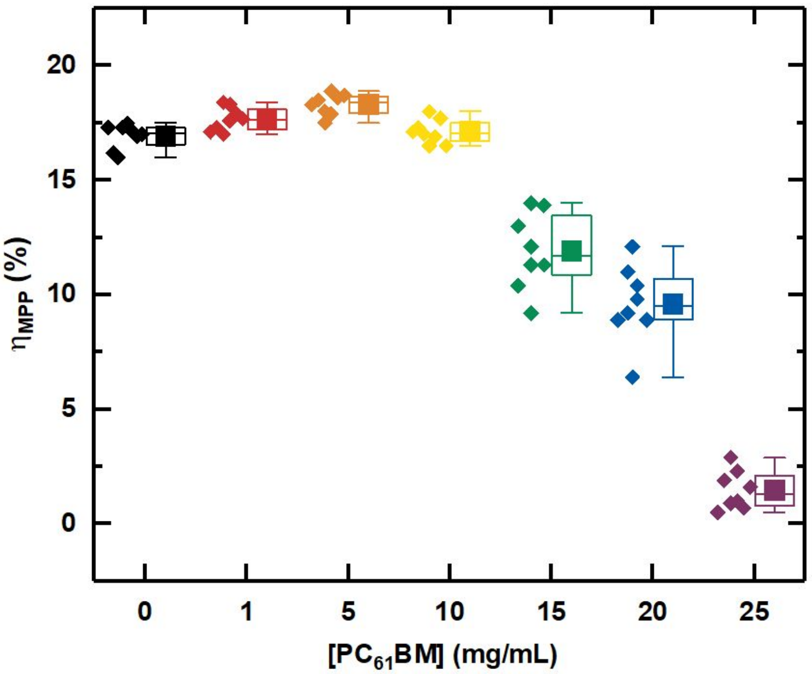

Figure S1. Steady-state efficiencies of n-i-p devices fabricated using varying concentrations of PCBM in the precursor solution.

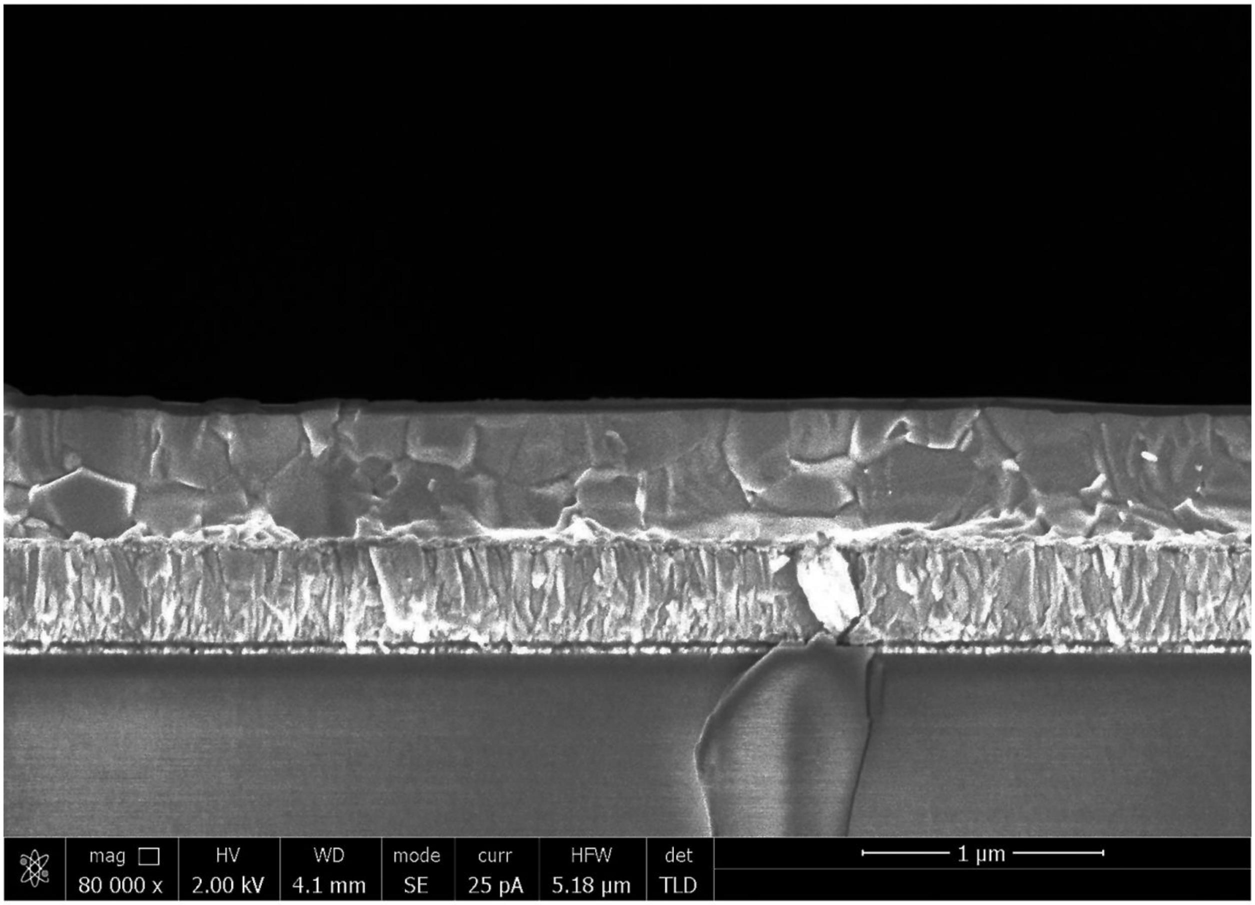

Figure S2: Cross-sectional SEM of a p-i-n device fabricated with 25 mg/mL PCBM in the BA/toluene perovskite precursor solution.

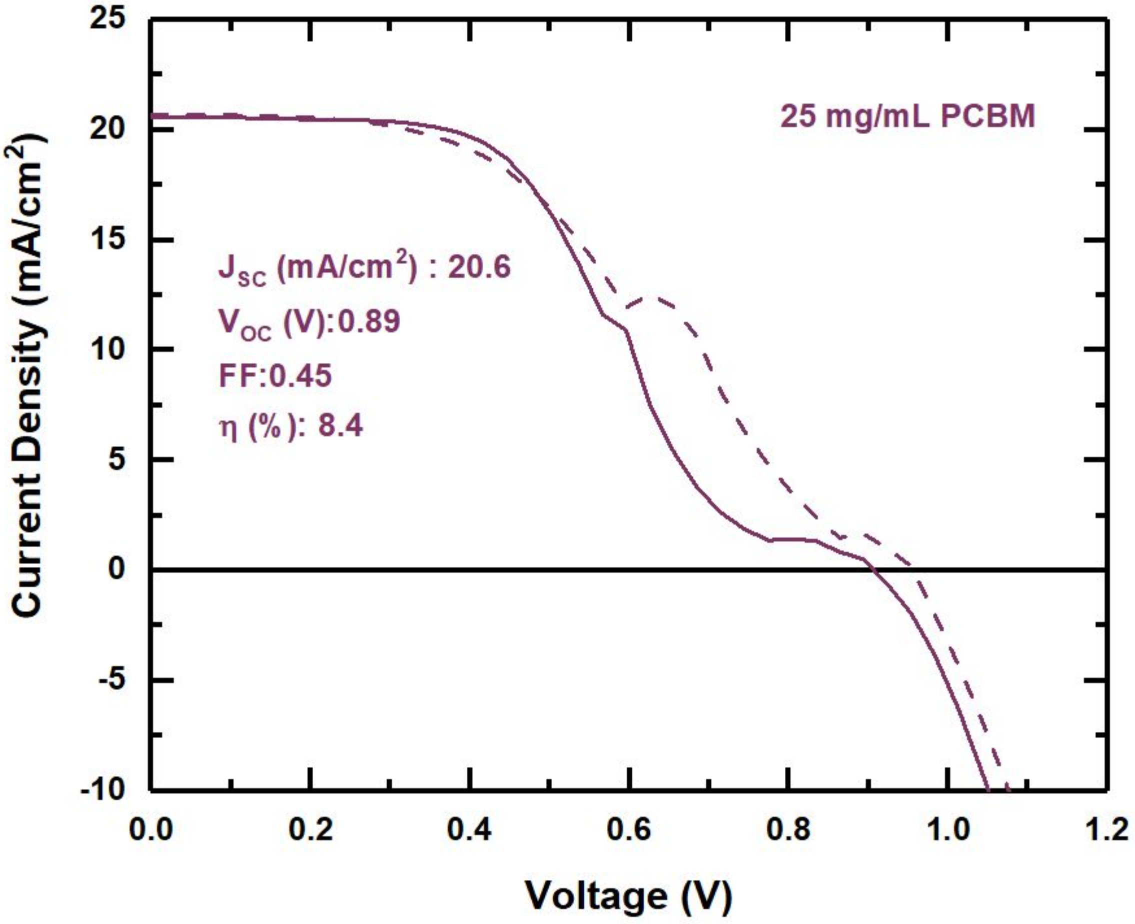

Figure S3. Current-voltage characteristics of champion n-i-p device made with 25 mg/mL of PCBM in the perovskite precursor solution.

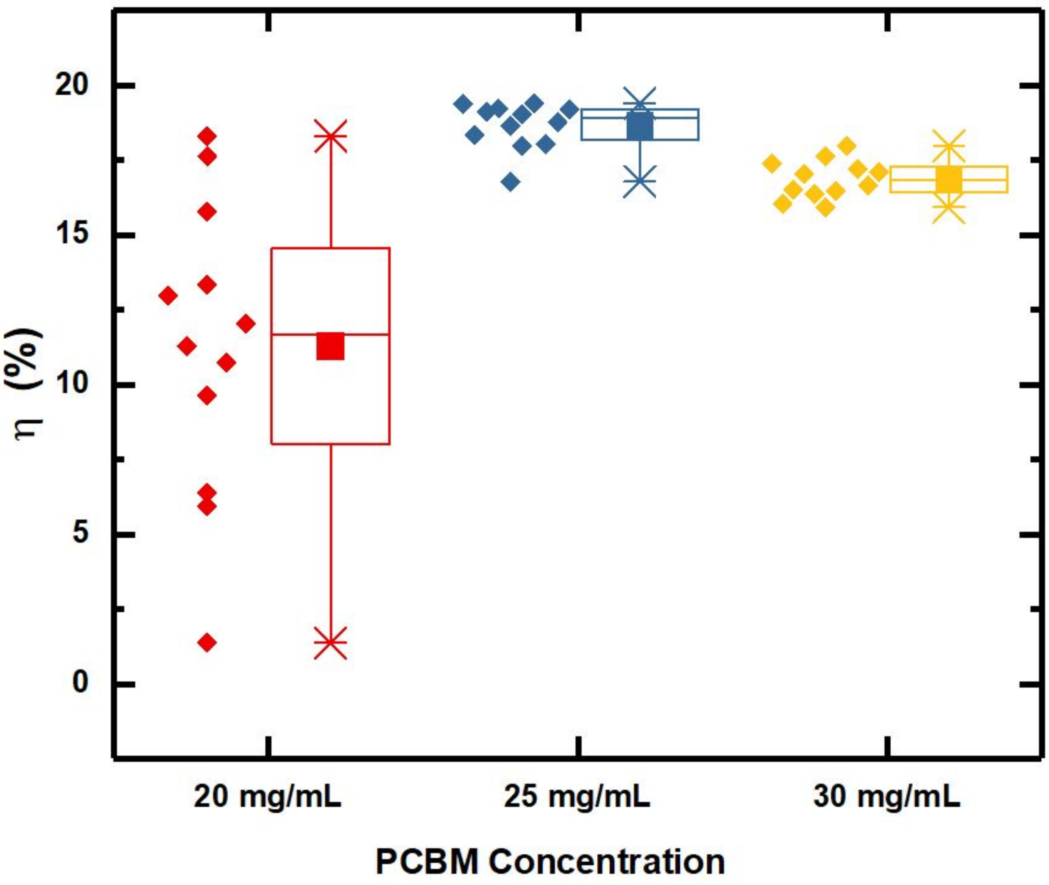

Figure S4. Efficiency distribution over p-i-n devices made with PCBM concentrations which cause visible stratification. Each data point represents one device (total 12) Whiskers depict the 25th to 75th percentile, with points outside this range as outliers.

References:

(1) Noel, N. K.; Habisreutinger, S. N.; Wenger, B.; Klug, M. T.; Horantner, M. T.; Johnston, M. B.; Nicholas, R. J.; Moore, D. T.; Snaith, H. J. A Low Viscosity, Low Boiling Point, Clean Solvent System for the Rapid Crystallisation of Highly Specular Perovskite Films. *Energy Environ. Sci.* **2017**, *10* (1), 145–152. https://doi.org/10.1039/C6EE02373H.

(2) Zhou, Z.; Wang, Z.; Zhou, Y.; Pang, S.; Wang, D.; Xu, H.; Liu, Z.; Padture, N. P.; Cui, G. Methylamine-Gas-Induced Defect-Healing Behavior of CH 3 NH 3 PbI 3 Thin Films for Perovskite Solar Cells. *Angew. Chemie Int. Ed.* **2015**, *54* (33), 9705–9709. https://doi.org/10.1002/anie.201504379.

(3) Noel, N. K.; Congiu, M.; Ramadan, A. J.; Fearn, S.; McMeekin, D. P.; Patel, J. B.; Johnston, M. B.; Wenger, B.; Snaith, H. J. Unveiling the Influence of PH on the Crystallization of Hybrid Perovskites, Delivering Low Voltage Loss Photovoltaics. *Joule* **2018**, *1* (2), 328–343. https://doi.org/10.1016/j.joule.2017.09.009.

(4) Anaraki, E. H.; Kermanpur, A.; Steier, L.; Domanski, K.; Matsui, T.; Tress, W.; Saliba, M.; Abate, A.; Gratzel, M.; Hagfeldt, A.; Correa-Baena, J.-P. Highly Efficient and Stable Planar Perovskite Solar Cells by Solution-Processed Tin Oxide. *Energy Environ. Sci.* **2016**, *9* (10), 3128–3134. https://doi.org/10.1039/C6EE02390H.

(5) Habisreutinger, S. N.; Wenger, B.; Snaith, H. J.; Nicholas, R. J. Dopant-Free Planar n–i–p Perovskite Solar Cells with Steady-State Efficiencies Exceeding 18%. *ACS Energy Lett.* **2017**, *2* (3), 622–628. https://doi.org/10.1021/acsenergylett.7b00028.
